# Lifelong learning dimensions and their associations with late-life cognitive decline: moderating roles of socioeconomic status and early life education

**DOI:** 10.3389/fpsyg.2025.1729306

**Published:** 2025-11-11

**Authors:** Feiran Zheng

**Affiliations:** School of Ethnology and Sociology, Minzu University of China, Beijing, China

**Keywords:** lifelong learning, cognitive decline, older adult health, socioeconomic status, early life education, moderating role, cognitive reserve

## Abstract

**Introduction:**

Amidst the global wave of population aging, safeguarding cognitive health in older adults is a pressing public health issue. However, the key components of lifelong learning and whether its benefits apply universally across social backgrounds remain unclear. This study aimed to identify distinct dimensions of lifelong learning and to test their effects on subjective cognitive function, as well as the moderating role of socioeconomic background.

**Methods:**

We conducted a cross-sectional survey of 278 Chinese older adults aged 60 and above. Exploratory factor analysis was used to delineate the dimensions of lifelong learning. Hierarchical regression analysis was then employed to assess the predictive effects of these dimensions on subjective cognitive function and the moderating effects of socioeconomic status (SES) and early-life education (ELE).

**Results:**

Two distinct dimensions were identified: “information-driven cognitive engagement” and “social interaction and experiential learning.” Only the former, characterized by cognitively challenging activities, showed a significant positive predictive effect on subjective cognitive function (*β* = 0.143, *p* = 0.017). Crucially, neither SES nor ELE significantly moderated this relationship.

**Discussion:**

The findings suggest that the cognitive benefits of challenging learning activities are broadly universal, transcending socioeconomic and educational divides. This “equitable benefit” provides strong empirical evidence for policy shifts from encouraging generalized “participation” to promoting inclusive and deep “cognitive engagement,” thereby fostering fairer and more effective cognitive health promotion strategies for older adults.

## 1 Introduction

### 1.1 Cognitive health challenges in global aging

Human society is entering an era of advanced aging at an accelerated pace. The World Health Organization (WHO) predicts that by 2050, the global population aged 60 and above will double to 2.1 billion (Reynolds 3rd et al., 2022). Amidst this profound demographic shift, maintaining cognitive function has become a core issue vital for the quality of life of billions of older adults and for sustainable societal development. Cognitive decline is not a trivial matter; it represents a continuum from subjective cognitive decline (SCD) to mild cognitive impairment (MCI), potentially culminating in dementia (Aarsland et al., 2021; Jiang et al., 2025; Ma et al., 2025). Against the backdrop of repeated setbacks in drug development, academic attention is increasingly focusing on non-pharmacological interventions, seeking “strategies” that can safeguard cognitive function (Zou et al., 2025).

Among numerous strategies, lifelong learning (LLL)—encompassing all purposeful learning activities throughout an individual’s life aimed at enhancing knowledge, skills, and abilities—shows immense potential. It is underpinned by robust theoretical frameworks: cognitive reserve theory posits that sustained cognitive activity builds more efficient and resilient neural networks to resist age- or pathology-related damage (Nogueira et al., 2022; Baciu et al., 2021), while neuroplasticity theory confirms that even in older age, the brain can still form new neural connections through learning and experience (Marzola et al., 2023). Extensive empirical research, including landmark longitudinal studies and randomized controlled trials such as the “Synapse Project,” has also consistently demonstrated the positive association of cognitively challenging learning activities on delaying cognitive decline (Uno et al., 2025).

However, despite the consensus that “learning is beneficial,” this broad conclusion may mask two more critical and pressing underlying questions, which form the impetus for our current research.

### 1.2 The first knowledge gap: opening the “black box” of lifelong learning—which type of learning is more influential?

A core limitation of current research lies in its tendency to treat lifelong learning as a homogeneous, overarching “black box.” Many studies use a summative score to measure learning participation, and their policy recommendations often remain at the vague level of “encouraging older adults to learn more.” However, the cognitive demands of different learning activities vary significantly: actively acquiring a complex digital photography skill is clearly distinct in its cognitive processing from participating in a relaxed social book club. Lumping them together, much like examining “exercise is beneficial for health” without differentiating between “walking” and “high-intensity interval training,” risks obscuring the “active ingredients” truly driving cognitive benefits.

The academic literature offers various frameworks for classifying lifelong learning, often distinguishing by formality (Laal, 2011) or purpose (Yamashita et al., 2017). The breadth of these activities is further illustrated by systematic reviews (Thwe and Kalman, 2024). However, these classifications typically rely on theoretical distinctions or aggregate diverse activities, often overlooking the specific “active ingredients” for cognitive health. Moreover, in the context of “technological aging,” the rise of digital learning highlights the need to differentiate between online and offline modalities and their distinct cognitive demands. Much existing research still treats lifelong learning as a monolithic concept, limiting our understanding of how different types of engagement differentially impact cognitive function.

In recent years, the landscape of lifelong learning for older adults has been profoundly reshaped by the digital revolution. The rise of “technological aging” or “digital aging” highlights how information and communication technologies (ICTs), such as smartphones and the internet, have become increasingly important to the lives of the elderly (Czaja and Lee, 2007). This digital shift has bifurcated learning opportunities into two distinct pathways: traditional, offline activities and modern, online engagement. Traditional learning often occurs in physical spaces and emphasizes social interaction. In contrast, online learning is typically self-directed, information-driven, and requires a different set of cognitive skills, including digital literacy, online navigation, and critical evaluation of information (Cotten et al., 2012). Indeed, some scholars argue that interaction with technology itself constitutes a form of experiential learning; for instance, seemingly recreational activities like game-playing on touchscreen devices have been identified as effective learning resources that encourage the adoption of new technologies (Oppl and Stary, 2020).

While existing literature extensively discusses the general benefits of lifelong learning, there remains a gap in empirically distinguishing the cognitive outcomes of these online versus offline modalities. It is plausible that the active, problem-solving nature inherent in navigating the digital world for information presents a more potent form of cognitive stimulation than more passive, socially oriented learning. This study, therefore, aims to explore whether distinct dimensions of lifelong learning, potentially aligning with this online/offline divide, emerge from older adults’ activities and whether differentially relate to cognitive function.

Our study addresses this by empirically unveiling the underlying structure of lifelong learning activities through exploratory factor analysis, rather than imposing pre-defined categories. This data-driven approach allows us to identify distinct learning dimensions, moving beyond broad classifications to answer ‘which type of learning is more significantly beneficial.’ This offers a more refined understanding and a level of detail often missing in prior studies focused on general participation.

Therefore, the first core objective of this study is to “open the black box” of lifelong learning, moving from the broad question of “whether to learn is effective” to the precise question of “which type of learning is more effective.” We hypothesize that not all learning activities contribute equally to building cognitive reserve. Activities requiring active information processing, logical reasoning, and problem-solving likely offer significantly greater cognitive protection than those primarily serving social or recreational needs. Based on this, we propose our first core hypothesis:

Hypothesis 1 (*H1*): Distinct dimensions of lifelong learning will differentially relate to cognitive function in older adults, with dimensions characterized by higher cognitive challenge demonstrating a stronger predictive association.

### 1.3 The second knowledge gap: testing the equity of cognitive benefits—for whom are the learning dividends?

Having identified the significant types of learning, a more socio-scientifically profound question arises: Are these cognitive benefits a universal boon accessible to all, or do they exacerbate existing socioeconomic advantages through a “Matthew effect”^1^? This is a critical issue concerning social equity (Merton, 1968).

On one hand, socioeconomic status (SES) and early-life education (ELE), as individuals’ crucial social resources and cognitive “initial capital,” likely influence their opportunities, motivation, and efficiency in lifelong learning (Kirkbride et al., 2024; Maehler et al., 2025; Morris et al., 2021). Individuals with higher SES and ELE possess more resources to engage in high-quality learning, and may also have mastered more effective learning strategies, enabling them to reap higher returns from their “cognitive re-investment” in older age. This logic points to the “accumulated advantage” hypothesis, suggesting that the cognitive dividends of lifelong learning might disproportionately favor socially advantaged groups, thereby widening health disparities (Li et al., 2025).

However, an alternative possibility exists: lifelong learning, as a powerful cognitive stimulus, could serve a broadly supportive role. The magnitude of its benefits might primarily depend on the cognitive challenge of the learning activity itself, rather than the participant’s background. Furthermore, for disadvantaged groups with relatively lower early-life cognitive reserve, later-life learning might play an even more crucial “compensatory” role.

Currently, empirical evidence regarding the moderating roles of SES and ELE in the relationship between lifelong learning and cognitive health remains inconsistent and lacks specificity. Therefore, the second core objective of this study is to directly address this debate by constructing moderation models to systematically test the equity of lifelong learning’s cognitive benefits. We propose the following two competing hypotheses:

Hypothesis 2 (*H2*): Socioeconomic status (SES) will positively moderate the relationship between the effective dimension(s) of lifelong learning and subjective cognitive function, meaning that cognitive benefits will be more pronounced in higher SES groups (supporting the ‘accumulated advantage’ hypothesis).

Hypothesis 3 (*H3*): Early-life education (ELE) will positively moderate the relationship between the effective dimension(s) of lifelong learning and subjective cognitive function, meaning that cognitive benefits will be more pronounced in groups with higher ELE (supporting the ‘accumulated advantage’ hypothesis).

### 1.4 Innovation and significance of the present study

The innovation of this study lies in systematically addressing the aforementioned two knowledge gaps through a unified research framework. We are committed not only to identifying the “key targets” for delaying cognitive decline (i.e., which type of learning) but also to examining the “equity” of its societal benefits (i.e., for whom). Our study’s sample is drawn from China, and its findings will provide localized empirical evidence for understanding how older adults in a transitional Chinese society can leverage lifelong learning to combat cognitive aging challenges, especially given China’s vast urban–rural disparities, uneven regional development, and historical inequalities in educational opportunities, under which the aforementioned two core questions are particularly salient (Guo et al., 2021).

The findings of this study will hold dual value. Theoretically, they will refine cognitive reserve theory and offer new evidence for understanding the deeper mechanisms of health equity. Practically, they will provide a clear “crossroads” guide for public policy in an aging society: should we design additional, compensatory programs specifically for disadvantaged groups, or should resources be concentrated on developing and popularizing universally applicable, cognitively challenging learning projects? The answer to this question is crucial for guiding the fairest and efficient allocation of social resources to promote “healthy aging” for the entire population.

## 2 Methods

### 2.1 Research design

We employed a cross-sectional survey design. This design collects data from a representative sample of a target population at a specific point in time to describe variable distributions and analyze relationships between variables. Given our aim to explore the association patterns and moderating mechanisms among lifelong learning, socioeconomic status, early life education, and subjective cognitive function, rather than causal relationships, the cross-sectional design offers advantages of efficiency, economy, and ease of implementation. It is suitable for rapidly acquiring large datasets in exploratory research stages to test theoretical hypotheses.

### 2.2 Participants

Participants in this study included 278 Chinese older adults recruited through online channels and community outreach. All participants read and consented to an informed consent form before the study began, ensuring their voluntary involvement.

#### 2.2.1 Inclusion criteria

Age ≥ 60 years old.

Able to understand Mandarin or local dialects and complete the questionnaire with the assistance of family members or researchers.

Agreed to participate in the survey.

#### 2.2.2 Exclusion criteria

Self-reported diagnosis of mental illnesses (schizophrenia) or neurological disorders (Alzheimer’s disease, Parkinson’s disease) that impair cognitive function.

Severe visual or hearing impairments that hinder normal communication and understanding of the questionnaire content.

### 2.3 Measures

We employed a structured survey questionnaire, which was meticulously developed based on established theoretical frameworks and drawing upon items from relevant validated scales, while carefully adapting them to the specific Chinese cultural context and the characteristics of older adults. This approach aimed to ensure both scientific rigor and cultural appropriateness. The questionnaire includes the following modules:

Basic demographic information: Covers age, gender, ethnicity, marital status, place of residence, and living arrangements.
Early life education (ELE): We operationalized Early Life Education (ELE Score) by quantifying the “highest completed education level,” coding it from 1 to 4 points (1 = “primary school and below” to 4 = “junior college and above”), with higher scores indicating higher levels of formal early education. This is a widely accepted proxy for cognitive reserve and access to lifelong learning opportunities in epidemiological studies.
Socioeconomic status (SES): We quantified Socioeconomic Status (SES Score) using multiple indicators: “occupation before retirement or current occupation” and “current average monthly total household income.” We standardized both occupation (1–6 points) and income (1–5 points) and summed them to obtain a continuous composite SES score. This multi-dimensional approach enhances the robustness of the SES measure by capturing both occupational prestige and economic resources.
Lifelong learning participation (LLL): This served as the core independent variable. The scale included 10 items carefully selected to capture a broad spectrum of lifelong learning activities. The selection was guided by existing literature on adult learning and engagement (Laal, 2011; Yamashita et al., 2017) and adapted to represent activities commonly undertaken by older adults in the Chinese context. These items were chosen to encompass diverse modalities, specifically aiming to represent both information-driven cognitive engagement (e.g., reading, using electronic devices) and social/experiential learning (e.g., socializing, visiting cultural venues), reflecting the multi-dimensional nature of lifelong learning as conceptualized in the field. We used a 5-point Likert scale (1 = never, 5 = daily). The total Lifelong Learning Participation score (LLL Score) was the average of the 10 item scores. The scale’s internal consistency reliability (Cronbach’s *α*) for this scale was 0.89 in the study sample, indicating good reliability, which supports the coherence of the selected items. Further empirical validation of these proposed dimensions was provided by the exploratory factor analysis conducted in our results section (Section 3.3).
Subjective cognitive function (SCF): This was the core dependent variable. The scale included eight items, specifically adapted from the conceptual framework and common item structures found in established subjective cognitive scales, such as the Everyday Cognition (ECog) scale (Farias et al., 2008) and other self-report measures of cognitive complaints in older populations. The adaptation focused on capturing participants’ self-perceived changes in key cognitive domains—memory, learning ability, attention, executive function, and language ability—compared to 5 years prior. This emphasis on self-reported decline aligns with the clinical understanding of subjective cognitive decline (SCD) as a potential early indicator of cognitive impairment. We used a 5-point Likert scale (1 = always, 5 = never). For easier interpretation, we reverse-coded the scores, so higher scores indicated better self-perceived cognitive function (i.e., fewer cognitive complaints). The total Subjective Cognitive Function score (SCF Score) was the average of the eight item scores. The scale’s Cronbach’s *α* for this scale was 0.92 in the study sample, indicating excellent reliability, thereby supporting its internal consistency within our sample.
Control variables: A broader range of covariates, including history of chronic diseases, frequency of physical exercise (Klotzbier and Schott, 2025), smoking history, alcohol consumption history, sleep duration, and vision and hearing status (Luo et al., 2024), were collected and initially considered for their potential influence on cognitive function. However, to maintain model parsimony and avoid overfitting given our sample size, only age and gender were ultimately included as demographic control variables in the main hierarchical regression models presented in Table 1.

**Table 1:** Hierarchical regression analysis results examining main effects of lifelong learning dimensions and moderation by SES and ELE (*N* = 278).

Variable (predictor)	Model 1: B (SE)	Model 2: B (SE)	Model 3: B (SE)	Model 4: B (SE)
Step 1: control variables				
Constant (intercept)	3.315 (0.033)***	3.327 (0.032)***	3.327 (0.032)***	3.316 (0.032)***
Age (centered)	0.003 (0.004)	−0.001 (0.004)	−0.001 (0.004)	0.002 (0.004)
Gender (0 = female, 1 = male)	0.004 (0.061)	−0.004 (0.059)	−0.004 (0.059)	0.001 (0.059)
Step 2: main effects				
Factor 1 (information-driven cognitive engagement)		0.076 (0.031)*	0.077 (0.031)*	0.074 (0.031)*
Factor 2 (social and experiential learning)		0.046 (0.030)	0.046 (0.030)	0.048 (0.030)
Step 3: moderator variables and interaction terms				
SES score (centered)			−0.021 (0.023)	
F1 × SES interaction			−0.016 (0.031)	
ELE score (centered)				0.019 (0.012)
F1 × ELE interaction				−0.010 (0.060)
Model statistics				
*R* ^2^	0.001	0.036	0.037	0.044
Adjusted *R*^2^	−0.006	0.018	0.016	0.021
*ΔR*^2^ (change from previous step)		0.035*	0.001	0.008
*F* (overall model)	0.17	2.05	1.83	1.95
*ΔF* (change from previous step)		3.333*	0.280	1.13

B, unstandardized regression coefficient; SE, standard error. *p < 0.05, **p < 0.01, ***p < 0.001.

### 2.4 Data collection procedures

Data for this study were collected in October 2025 using the online questionnaire platform “Wenjuanxing.” Researchers recruited 278 participants aged 60 and above by collaborating with community elderly care service centers and posting recruitment information in online social media groups. Recognizing the characteristics of older adults, the questionnaire allowed for completion with assistance from family members or others. Clear instructions were provided, requesting assistants to act solely as “readers” and “recorders” to ensure that answers genuinely reflected the older adults’ intentions. All participants reviewed and agreed to an electronic informed consent form before completing the questionnaire.

### 2.5 Data analysis strategy and statistical models

After cleaning, the collected data were imported into a Python 3.9 environment for statistical analysis using libraries such as pandas and statsmodels. The analysis steps included:

Descriptive statistics and correlation analysis: We used frequencies, percentages, means, and standard deviations to describe sample characteristics. We used Pearson correlation matrices to examine preliminary relationships among key variables.
Exploratory factor analysis (EFA): To investigate the internal structure of lifelong learning, we subjected the 10 items of the lifelong learning activities scale to exploratory factor analysis. We used principal component analysis for factor extraction and Varimax orthogonal rotation to achieve a clear factor structure.
Hierarchical regression analysis: To test the core hypotheses, we constructed hierarchical regression models with subjective cognitive function (SCF Score) as the dependent variable. All continuous variables introduced into the model (age and EFA-extracted factor scores) were mean-centered.
Model 1 (baseline model): Included only control variables (centered age, gender) to assess the explanatory power of basic variables.
Model 2 (main effects model): Built upon Model 1, adding the two EFA-extracted factors (“information-driven cognitive engagement” score, “Social Interaction and Experiential Learning” score) to test their main effects on subjective cognitive function.

- Moderation effect test: Building upon Model 2, we constructed two separate models, each incorporating an interaction term between the “information-driven cognitive engagement” factor score and the centered SES score, and the ELE score, respectively, to test Hypotheses H2 and H3.

All statistical tests used a significance level at *α = 0.05*.

## 3 Results

### 3.1 Sample demographics and variable descriptives

We ultimately analyzed 278 valid questionnaires. Table 2 presents the demographic characteristics of the sample. Participants’ average age was 68.73 years (SD = 6.54, range 60–80 years). Women (*N* = 153, 55.04%) slightly outnumbered men (*N* = 125, 44.96%). In terms of ethnicity, Han Chinese constituted the vast majority (*N* = 248, 89.21%), with ethnic minorities accounting for 10.79% (*N* = 30). Early life education levels showed considerable heterogeneity, with junior high school (37.77%) and primary school or below (35.25%) as the predominant educational backgrounds, which aligns with the overall educational structure of China’s current older adult population.

**Table 2:** Sample demographic characteristics (*N* = 278).

Feature	Category	Frequency (*N*)	Percentage (%)
Gender	Male	125	44.96%
	Female	153	55.04%
Ethnicity	Han	248	89.21%
	Ethnic minority	30	10.79%
Education level (ELE)	Primary school and below	98	35.25%
	Junior high school	105	37.77%
	High school/secondary vocational/technical school	55	19.78%
	Junior college and above	20	7.19%
Age	Mean (SD)	68.73 (6.54)	
	Min–Max	60–80	

Table 3 presents the descriptive statistics for the main study variables. The mean Lifelong Learning Participation score (LLL Score) was 3.15 (SD = 0.88), indicating a moderate level of overall learning engagement among older adults. The mean Subjective Cognitive Function score (SCF Score) was 3.48 (SD = 0.75), with a wide range of scores, suggesting considerable individual differences in subjective cognitive perceptions within the sample.

**Table 3:** Descriptive statistics of key study variables.

Variable	Mean (M)	Standard deviation (SD)	Minimum	Maximum
Lifelong learning participation (LLL score)	3.15	0.88	1.00	5.00
Subjective cognitive function (SCF score)	3.48	0.75	1.25	5.00

### 3.2 Correlation analysis

**Figure 1:**
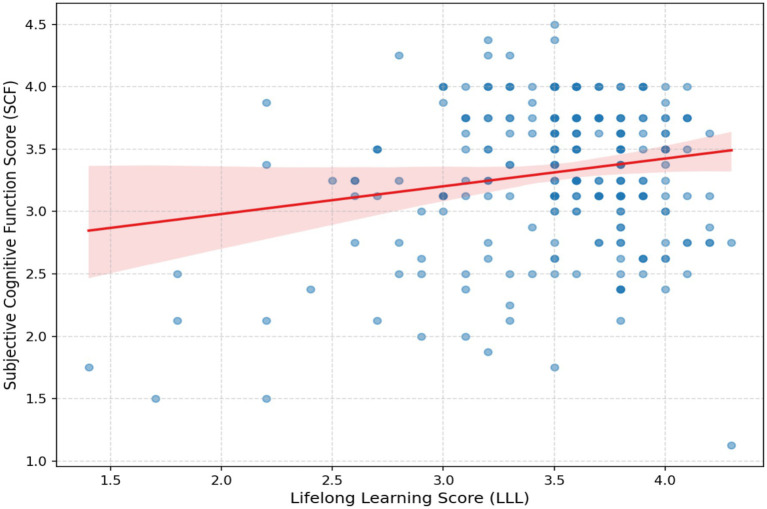
Scatter plot and fitted line of lifelong learning score (LLL) and subjective cognitive function score (SCF).

To explore relationships among variables, we conducted Pearson correlation analysis (see Table 4). Results showed a moderate and significant positive correlation between Lifelong Learning Participation (LLL) and Subjective Cognitive Function (SCF) (*r* = 0.48, *p* < 0.001), providing initial strong support for Hypothesis 1. This correlation suggests that older adults who more frequently engage in various learning activities tend to report better subjective cognitive states. As Figure 1 shows, the data points clearly trend upward to the right, and the linear regression line distinctly illustrates this positive relationship.

**Table 4:** Pearson correlation matrix of key variables.

Variable	LLL score	SCF score	SES score	ELE score
LLL score	1			
SCF score	0.48*	1		
SES score	0.51*	0.39*	1	
ELE score	0.42*	0.35*	0.63*	1

*p < 0.001.

Socioeconomic Status (SES) and Early Life Education (ELE) also showed significant positive correlations with SCF (*r* = 0.39, *p* < 0.001; *r* = 0.35, *p* < 0.001), consistent with cognitive reserve theory. Additionally, LLL, SES, and ELE exhibited significant positive correlations among themselves, indicating that individuals with higher education and socioeconomic status tend to participate more in lifelong learning, which aligns with theoretical expectations.

### 3.3 Structural dimensions of lifelong learning: exploratory factor analysis

To delve into the internal structure of the lifelong learning concept, we performed an exploratory factor analysis (EFA) on the ten items comprising the lifelong learning scale. First, data suitability tests yielded ideal results: the KMO measure of sampling adequacy was 0.76 (>0.7), and Bartlett’s test of sphericity was highly significant (*χ^2^* = 782.82, *p* < 0.001), indicating the data were well-suited for factor analysis.

**Figure 2:**
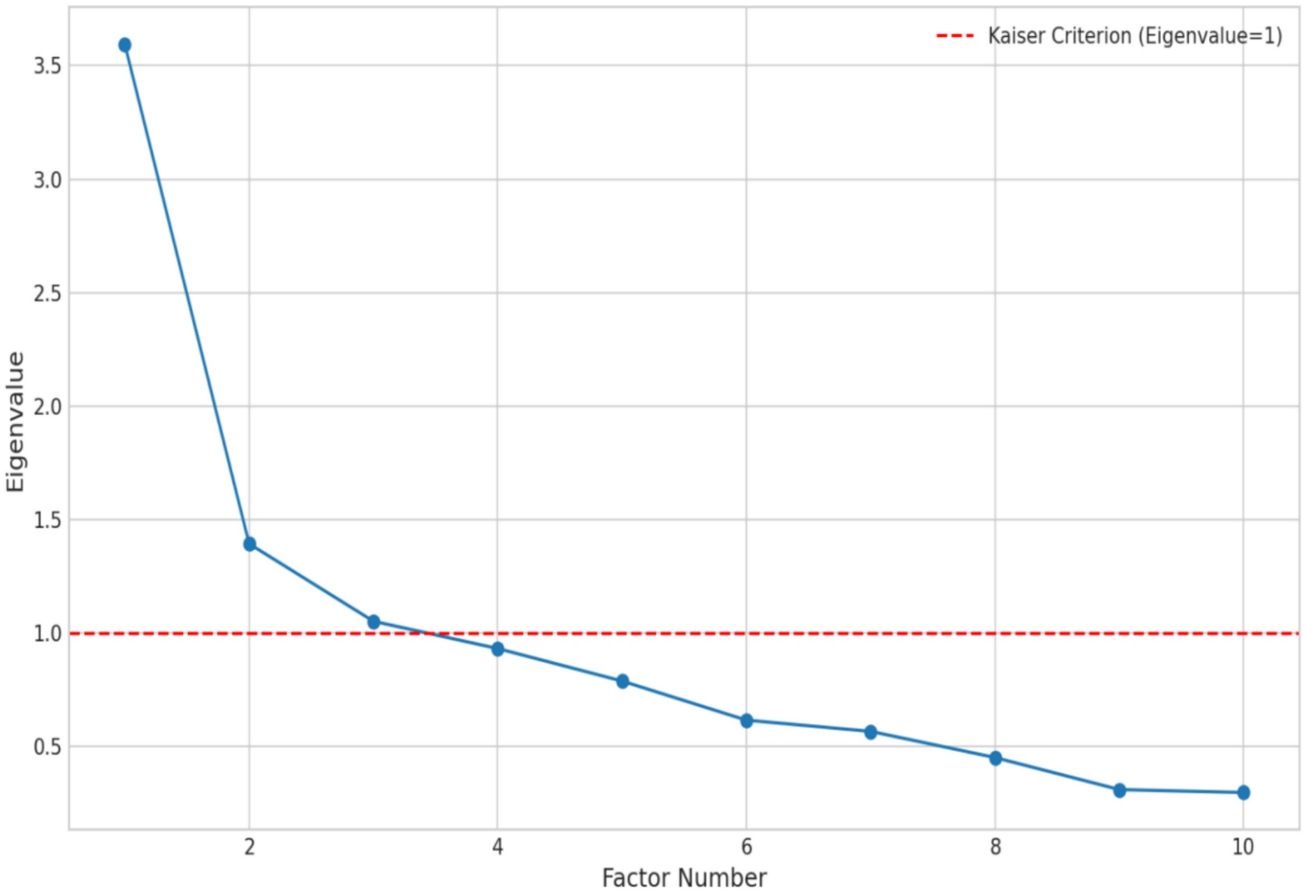
Factor analysis plot of lifelong learning activities.

Subsequently, we used principal component analysis to extract common factors. The number of extracted factors primarily followed Kaiser’s criterion (retaining factors with eigenvalues greater than 1) and the Scree Test. As Figure 2 shows, the scree plot exhibited a distinct “elbow” after the second factor, consistent with the eigenvalue test result (exactly two factors had eigenvalues greater than 1). Thus, we ultimately decided to extract two common factors.

To obtain a more interpretable factor structure, we applied Varimax orthogonal rotation to the factor loading matrix. The rotated factor loading matrix clearly revealed two distinct item clusters, as shown in Table 5. Based on the core characteristics of each cluster, we directly named these two factors “information-driven cognitive engagement” and “social interaction and experiential learning.”

**Table 5:** Rotated factor loading matrix of lifelong learning scale exploratory factor analysis (*N* = 278).

Lifelong learning activity	Factor 1 (information-driven cognitive engagement)	Factor 2 (social interaction and experiential learning)
Reading	**0.803**	0.083
Using digital devices	**0.673**	0.226
Watching educational TV	**0.686**	0.205
Playing puzzles	**0.454**	0.203
Learning new skills	**0.409**	0.290
Socializing with friends	0.068	**0.701**
Visiting cultural venues	0.184	**0.647**
Deep discussions	0.301	**0.552**
Attending courses	0.220	**0.532**
Traveling	0.181	0.202
Variance explained (%)	21.50%	17.63%
Cumulative variance explained (%)	21.50%	39.13%

Items with an absolute factor loading greater than 0.40 are considered to have significant loadings on that factor and are bolded.

As Table 5 illustrates, the structure of the two factors is very clear. The “information-driven cognitive engagement” factor primarily covers activities requiring active information processing and independent thinking, such as reading, using electronic devices, and watching educational TV programs. The “social interaction and experiential learning” factor, conversely, includes activities like socializing with friends and visiting cultural venues, where learning occurs through interpersonal interaction and direct experience. These two factors collectively explain 39.13% of the total variance.

This finding indicates that “lifelong learning” is not a singular concept but comprises two distinct types of activities. This offers a more refined perspective for understanding how lifelong learning influences cognitive function and lays the groundwork for subsequent mechanism exploration.

The mathematical model for exploratory factor analysis is shown in Equation 1:


$$X_{j}=a_{j1}F_{1}+a_{j2}F_{2}+\ldots +a_{\mathrm{jm}}F_{m}+\epsilon_{j}$$ (1)

where $X_{j}$ is the j observed variable (i.e., one of the 10 learning activities), $F_{1}$ is the i common factor (m=2), $a_{\mathrm{ji}}$ is the loading of the j variable on the i factor, $\epsilon_{j}$ is the unique factor for the j variable, representing the part not explained by the common factors.

**Figure 3:**
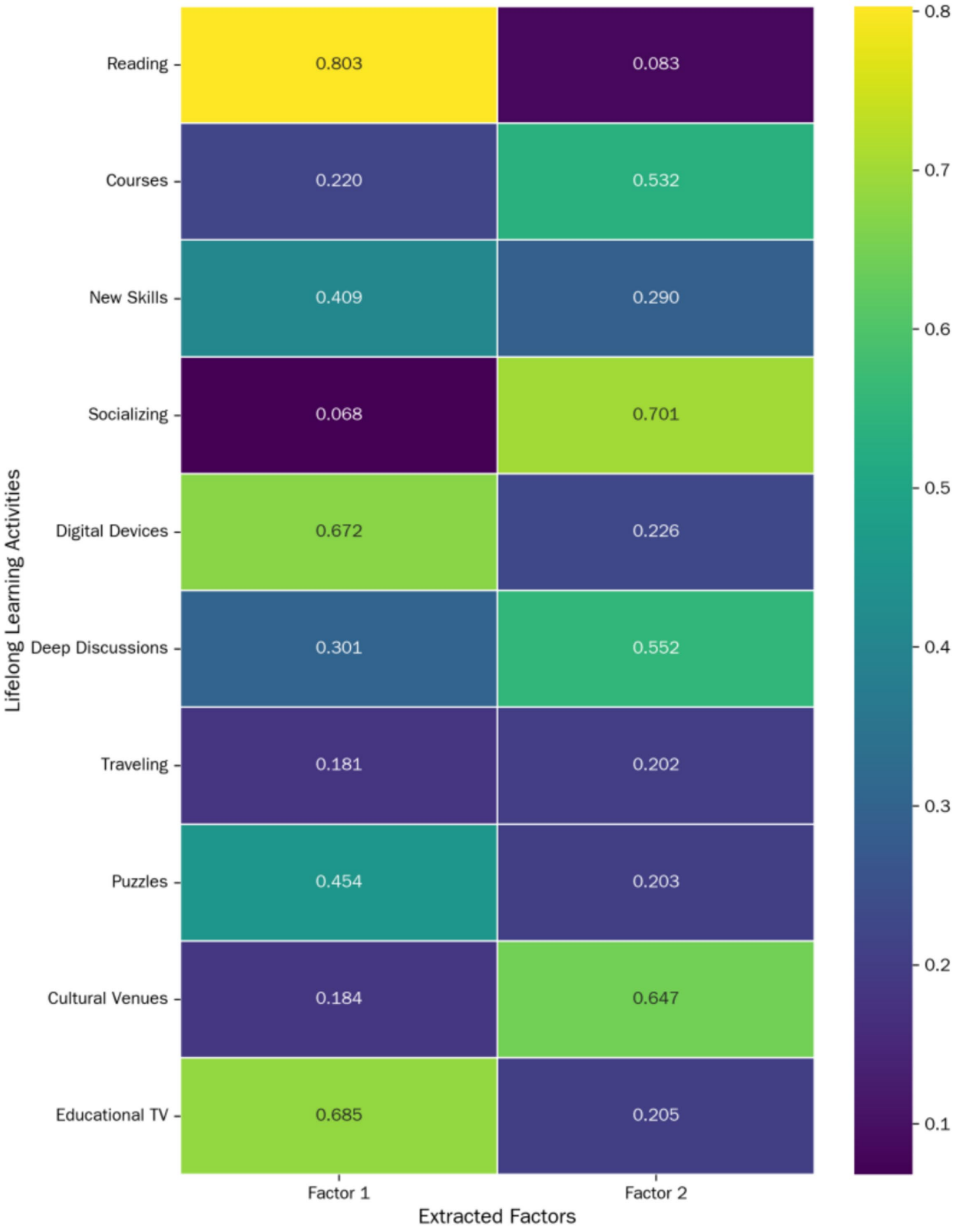
Rotated factor loading heatmap of lifelong learning activities.

Figure 3 visually represents the distribution of factor loadings for each item across the two factors.

### 3.4 Main hypothesis testing: hierarchical regression analysis

**Figure 4:**
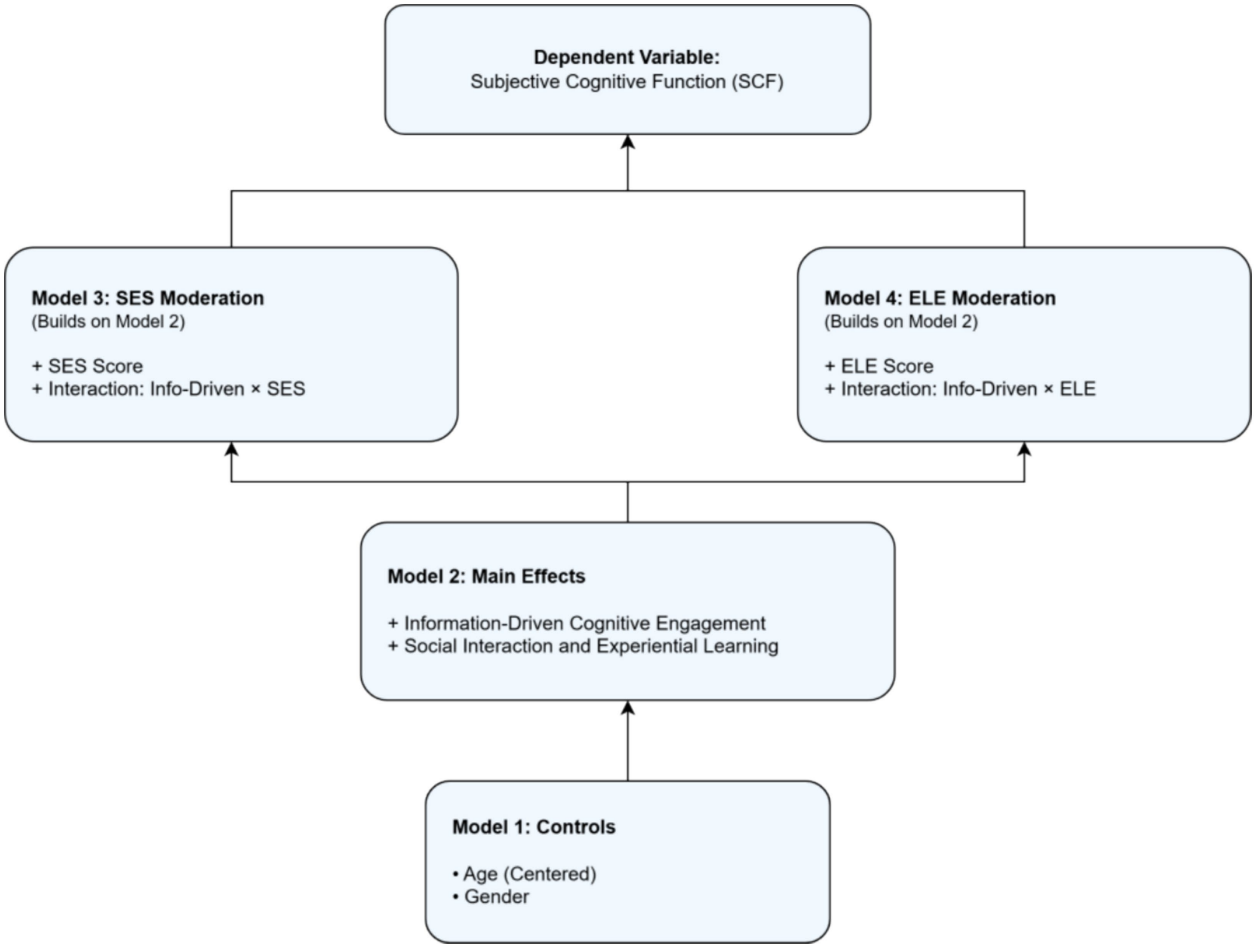
Visual representation of the hierarchical regression models.

To test the three core hypotheses of this study, we employed hierarchical regression analysis. Before analysis, all continuous independent variables (age, SES scores, ELE scores, and scores for the two lifelong learning factors) were mean-centered to mitigate potential multicollinearity issues. The analysis proceeded in the following steps, which are also visually summarized in Figure 4:

Model 1 (baseline model): This initial model included only the demographic control variables (centered age and gender) to assess their baseline impact on SCF.
Model 2 (main effects model): Building upon Model 1, we added the two lifelong learning dimensions (‘information-driven cognitive engagement’ and ‘social interaction and experiential learning’) to test their main effects and evaluate our first hypothesis (*H1*).
Model 3 (SES moderation model): To test for the moderating role of socioeconomic status (*H2*), this model was built upon Model 2 by adding the main effect of the centered SES score and, crucially, the interaction term between ‘information-driven cognitive engagement’ and the SES score.
Model 4 (ELE moderation model): Similarly, to test for the moderating role of early life education (H3), this model was built upon Model 2 by adding the main effect of the centered ELE score and its corresponding interaction term with ‘information-driven cognitive engagement’.

The detailed results for all hierarchical regression models are presented in Table 1.

#### 3.4.1 Differential main effects of lifelong learning dimensions (*H1* testing)

In the first step of the hierarchical regression (Model 1), only demographic control variables were included. Results showed that this model had very low explanatory power for subjective cognitive function (*R*^2^ *=* 0.001). In the second step (Model 2), after simultaneously adding the two lifelong learning dimensions (‘information-driven cognitive engagement’ and ‘social interaction and experiential learning’), the model’s overall explanatory power significantly increased (*ΔR*^2^ = 0.035, *ΔF* = 3.333, *p* < 0.05).

**Figure 5:**
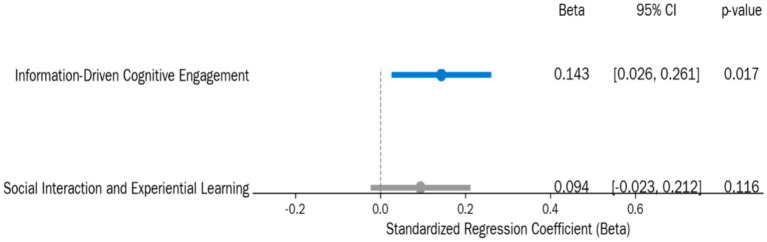
Standardized regression coefficient forest plot for two dimensions of lifelong learning on subjective cognitive function.

An examination of the regression coefficients for the two lifelong learning dimensions in Model 2 revealed significant differential effects. To provide a comprehensive and transparent comparison of the relative importance of these two core dimensions, we integrated the standardized regression coefficients (Beta), 95% confidence intervals (95% CI), and *p*-values into Figure 5.

As clearly illustrated in Figure 5, ‘information-driven cognitive engagement’ demonstrated a significant positive predictive power for subjective cognitive function (*β* = 0.143, *p* = 0.017), with its 95% confidence interval (0.026, 0.261) entirely above zero. In contrast, the predictive effect of ‘social interaction and experiential learning’ was not significant (*β* = 0.094, *p* = 0.116), and its confidence interval (−0.023, 0.212) clearly straddled zero. This indicates that, after controlling for age and gender, elders who engaged more in information-driven cognitive activities reported better subjective cognitive function.

The non-significant predictive role of ‘social interaction and experiential learning’ (*β* = 0.046, SE = 0.030, *p* > 0.05) further supported this distinction. This finding provides partial support for Hypothesis 1 (H1): lifelong learning does not uniformly influence cognitive function; rather, its specific dimension—‘information-driven cognitive engagement’—demonstrates a significant predictive effect.

Having established that “information-driven cognitive engagement” is a key active ingredient protecting subjective cognitive function, the next critical question is whether its benefits are universally accessible or are conditional upon an individual’s socioeconomic background. This leads us to the examination of our second and third hypotheses.

#### 3.4.2 Moderating effects of SES and ELE (H2 and H3 testing)

To test the moderating effects of socioeconomic status (H2) and early life education (H3), we built upon Model 2 by constructing Model 3 (to test SES moderation) and Model 4 (to test ELE moderation), respectively.

Hypothesis 2 testing (SES moderation effect): To test Hypothesis H2, we introduced the main effect of socioeconomic status (SES) and its interaction term with the core predictor ‘information-driven cognitive engagement’ (F1) into Model 3. As shown in Table 1, compared to Model 2, the increase in explanatory power for Model 3 was not significant (*ΔR^2^* = 0.001, *ΔF* = 0.280, *p* > 0.05). The unstandardized regression coefficient for the interaction term ‘F1 × SES’ was −0.016 (SE = 0.031, *p* = 0.598), did not reach statistical significance. This indicates that the data do not support the moderating role of socioeconomic status in the relationship between ‘information-driven cognitive engagement’ and subjective cognitive function. Thus, Hypothesis H2 was not supported by the data from this study.
Hypothesis 3 testing (ELE moderation effect): To test Hypothesis H3, we introduced the main effect of early life education (ELE) and its interaction term with the core predictor ‘information-driven cognitive engagement’ (F1) into Model 4. Similar to Model 3, the increase in explanatory power for Model 4 compared to Model 2 was also not significant (*ΔR^2^* = 0.008, *ΔF* = 1.13, *p* > 0.05). The unstandardized regression coefficient for the interaction term ‘F1 × ELE’ was −0.010 (SE = 0.060, *p* = 0.862), also did not reach statistical significance. This indicates that the data do not support the moderating role of early life education in the relationship between ‘information-driven cognitive engagement’ and subjective cognitive function. Consequently, Hypothesis H3 was also not supported by the data from this study.

In summary, the hierarchical regression analysis results indicate that ‘information-driven cognitive engagement’ has a significant positive predictive effect on subjective cognitive function in older adults, while ‘social interaction and experiential learning’ does not. Furthermore, the data from this study did not reveal significant moderating effects of socioeconomic status or early life education on the relationship between ‘information-driven cognitive engagement’ and subjective cognitive function.

## 4 Discussion

We conducted a cross-sectional survey of 278 Chinese older adults to systematically examine the associations with protecting cognitive function and explored the moderating mechanisms of socioeconomic status (SES) and early life education (ELE). Our core findings are twofold: first, we discovered that different dimensions of lifelong learning have varying impacts on cognitive function, with “information-driven cognitive engagement” showing a significant positive predictive effect; second, we found no significant moderating effects of SES and ELE on this relationship. This section will delve into these findings, discussing their theoretical implications, practical insights, limitations, and future directions.

### 4.1 The differentiated impact of lifelong learning: a digital aging perspective

The primary contribution of this study is the empirical identification of two distinct dimensions of lifelong learning and the discovery that only one—‘information-driven cognitive engagement’—significantly predicts better subjective cognitive function. Interpreted through the lens of technological aging, these two factors appear to represent a crucial distinction between modern, digitally driven learning (online) and traditional, socially embedded learning (offline).

‘Information-driven cognitive engagement’ (F1) primarily includes modern, digital activities such as using search engines and navigating online resources. These tasks are not passive; they require active cognitive processes like strategic planning, mental flexibility, and critical evaluation, effectively acting as a “cognitive workout” that aligns with cognitive reserve theory (Gkintoni et al., 2025). In sharp contrast, ‘social interaction and experiential learning’ (F2) embodies traditional, often offline, activities like attending classes or traveling. While crucial for social and emotional well-being, these activities may be less cognitively demanding in a direct, problem-solving sense. This distinction likely explains why F1, with its inherent problem-solving nature, showed a significant association with cognitive function while F2 did not.

Therefore, our findings do not diminish the value of social and experiential learning but rather specify its primary role. The key insight is that in the context of modern aging, it is the active, self-directed, and often digitally mediated pursuit of information that appears to be a particularly powerful ingredient in lifelong learning for maintaining cognitive health. This provides a crucial, evidence-based direction for designing more targeted and effective cognitive health interventions for older adults.

### 4.2 “Universal buffering”: an in-depth analysis of non-significant moderation effects

An equally important, and perhaps more sociologically important, contribution stems from the direct disconfirmation of our initial hypotheses (H2, H3): we found that the cognitive benefits derived from highly challenging learning are broadly universal.

Contrary to the “elite dividend” concern—that cognitive benefits might disproportionately favor the privileged—our data show this positive effect does not significantly vary by socioeconomic status (SES) or early-life education (ELE).

Far from being a failure of the study, it reveals a hopeful and valuable insight into health equity. This discovery compels us to think more deeply about traditional theoretical models and complex real-world situations. To this end, we propose several possible explanations:

### 4.3 Explanation one: statistical power limitations

This is a common and rigorous explanation for null findings. Detecting interaction effects (moderation) statistically requires a larger sample size than detecting main effects. Although our sample of 278 was sufficient for testing main effects, reliably detecting potentially true but small-effect-size moderation might require hundreds or even thousands of participants. Therefore, we cannot definitively claim that “no moderation effect absolutely exists”; a more precise statement is, “we did not find a significant moderation effect with the current sample size.” Future research should use an *a priori* power analysis to estimate the required sample size and conduct larger-scale data collection.

### 4.4 Explanation two: the “universal buffering” hypothesis

This offers a more positive and optimistic interpretation. This hypothesis suggests that lifelong learning, as a powerful form of cognitive stimulation, provides neuroprotective and cognitive enhancement effects so broadly fundamental and universal that its positive impact largely transcends or offsets background differences stemming from SES and ELE. Thus, regardless of an individual’s “initial capital” (ELE) and “current resources” (SES), as long as they initiate and persist in lifelong learning as a “cognitive investment,” they can expect to reap meaningful “cognitive returns.” If this explanation holds, it carries significant practical significance, implying that lifelong learning is a highly promising and equitable cognitive health intervention strategy capable of benefiting all social strata.

### 4.5 Explanation three: mismatched mechanisms of social factor influence

Our initial assumption was that SES and ELE would moderate the “effect” of learning, but their more likely mode of action is influencing the “opportunity” for learning. The correlation analysis results (Table 4) have already confirmed this: both SES and ELE show significant positive correlations with lifelong learning participation. This means that advantages in socioeconomic and educational background primarily manifest in making individuals more likely to participate in lifelong learning, rather than in gaining “better” outcomes once they participate. In the specific social context of China, given the increasing services provided to older adults in communities, coupled with assistance from their children, even some older adults who had limited schooling or currently face economic challenges can find learning opportunities (e.g., free community courses, help from children). Once they start learning, the benefits they derive may well be identical to those experienced by high-SES/ELE groups.

### 4.6 Theoretical and practical significance of the study

Despite the non-support of the moderation hypotheses, our findings still hold significant theoretical and practical value.

Theoretically, by differentiating the dimensions of lifelong learning, we refined the application of cognitive reserve theory. This suggests that not all cognitive activities equally build cognitive reserve; the “cognitive challenge” of the activity is key. Concurrently, the non-significant moderation effects challenge existing theories, prompting us to reconsider the distinct roles social structural factors might play in the “construction” and “utilization” stages of cognitive reserve.

Practically, a key implication is that the focus of efforts should perhaps not be on designing “different effect” learning programs for older adults of different backgrounds, but rather on eliminating barriers to ensure that older adults of all backgrounds can “equally participate” in cognitively challenging learning programs.

For policymakers: They should elevate the promotion of lifelong learning for older adults to a national strategy for addressing an aging society and building a “Healthy China” (Tohit and Haque, 2024). While increasing investment in community education and universities for older adults, attention should be paid to curriculum development, adding challenging and engaging courses (e.g., smartphone photography, basic programming, and historical critical thinking) rather than just recreational activities.

For community organizations: They should design and offer diverse, low-threshold, and accessible learning programs, particularly encouraging and assisting older adults with lower education and income to participate in “information-driven cognitive engagement” activities. For instance, they can organize mutual aid groups for smartphone use or host discussion sessions for easily understandable documentaries.

For families and individuals: They should encourage older family members to maintain habits of reading, thinking, and learning new skills. Helping them overcome fears of new technologies provides invaluable cognitive stimulation and emotional support.

### 4.7 Limitations and future directions of the study

This study’s methodological rigor was constrained by several key limitations:

First, the cross-sectional design prevents the establishment of causality. Second, all data were based on self-reports, which are susceptible to recall and social desirability biases. Third, assisted questionnaire completion by family members could have introduced unconscious influence. Fourth, the convenience sampling method may not fully represent the entire older adult population, potentially excluding the more isolated groups. Finally, our quantitative measures for complex concepts like SES and ELE are simplifications that may not capture deeper nuances such as educational quality or inherent learning abilities.

Based on these limitations, future research should aim for a more robust methodological framework. We recommend employing longitudinal designs to clarify the causal direction between learning and cognitive function; incorporating objective cognitive measures, such as neuropsychological tests, to validate the self-reported findings; utilizing randomized controlled trials (RCTs) to definitively assess intervention effects; and undertaking qualitative or mixed-methods research to offer a deeper, contextualized understanding of older adults’ learning experiences, particularly exploring the nuanced life-course roles of SES and ELE (Guo et al., 2022).

## 5 Conclusion

Through an empirical survey of 278 Chinese older adults, our study offers two clear and significant contributions to addressing the cognitive health challenges of an aging society.

First, this research opens the “black box” of lifelong learning, providing clear evidence that not all learning activities are equally beneficial for cognitive function. “Information-driven cognitive engagement,” characterized by higher cognitive challenge (e.g., reading, learning with electronic devices), is a key predictive factor against subjective cognitive decline. This provides a practical roadmap for future intervention strategies, shifting the focus from generalized participation to targeted cognitive investment.

Second, and perhaps more profoundly, our study reveals that these cognitive benefits are broadly universal and not significantly moderated by an individual’s socioeconomic status or early-life educational background. This robust evidence dispels concerns that cognitive health maintenance might exacerbate social inequalities, uncovering the significant potential of cognitively challenging learning as an inclusive and highly equitable public health strategy.

In summary, our findings offer a clear practical direction for public health policy in an aging society: we must not only encourage older adults to “live and learn” but also create conditions and lower barriers to actively guide them toward learning activities that genuinely exercise the mind and are challenging. By fostering a social environment that supports deep cognitive engagement, we can make a substantial contribution to extending the nation’s “healthy cognitive lifespan,” enhancing the quality of life for millions of older adults, and achieving the important goal of “healthy aging.”

## Data Availability

The original contributions presented in the study are included in the article/supplementary material, further inquiries can be directed to the corresponding author.

## Footnotes

^1^Matthew Effect: A sociological concept introduced by Robert K. Merton (1968), illustrating the phenomenon of “accumulated advantage” where those with initial advantages in resource and opportunity allocation tend to gain more, leading to a widening gap where “the rich get richer, and the poor get poorer.”

## References

[ref1] Aarsland D. Batzu L. Halliday G. M. Geurtsen G. J. Ballard C. Ray Chaudhuri K. . (2021). Parkinson disease-associated cognitive impairment. Nat. Rev. Dis. Primers 7:47. doi: 10.1038/s41572-021-00280-3, PMID: 34210995

[ref2] Baciu M. Banjac S. Roger E. Haldin C. Perrone-Bertolotti M. Lœvenbruck H. . (2021). Strategies and cognitive reserve to preserve lexical production in aging. Geroscience 43, 1725–1765. doi: 10.1007/s11357-021-00367-5, PMID: 33970414 PMC8492841

[ref3] Cotten S. R. Ford G. Ford S. Hale T. M. (2012). Internet use and depression among older adults. Comput. Human Behav. 28, 496–499. doi: 10.1016/j.chb.2011.10.021

[ref4] Czaja S. J. Lee C. C. (2007). The impact of aging on access to technology. Univ. Access Inf. Soc. 5, 341–349. doi: 10.1007/s10209-006-0060-x, PMID: 41159878

[ref5] Farias S. T. Mungas D. Reed B. R. Cahn-Weiner D. Jagust W. Baynes K. . (2008). The measurement of everyday cognition (ECog): scale development and psychometric properties. Neuropsychology 22, 531–544. doi: 10.1037/0894-4105.22.4.531, PMID: 18590364 PMC2877034

[ref6] Gkintoni E. Antonopoulou H. Sortwell A. Halkiopoulos C. (2025). Challenging cognitive load theory: the role of educational neuroscience and artificial intelligence in redefining learning efficacy. Brain Sci. 15:203. doi: 10.3390/brainsci15020203, PMID: 40002535 PMC11852728

[ref7] Guo J. Huang X. Dou L. Yan M. Shen T. Tang W. . (2022). Aging and aging-related diseases: from molecular mechanisms to interventions and treatments. Signal Transduct. Target. Ther. 7:391. doi: 10.1038/s41392-022-01251-0, PMID: 36522308 PMC9755275

[ref8] Guo M. Li M. Xu H. Stensland M. Wu B. Dong X. (2021). Age at migration and cognitive health among Chinese older immigrants in the United States. J. Aging Health 33, 709–720. doi: 10.1177/08982643211006612, PMID: 33847534 PMC8667381

[ref9] Jiang Q. Liu J. Huang S. Wang X. Y. Chen X. Liu G. H. . (2025). Antiageing strategy for neurodegenerative diseases: from mechanisms to clinical advances. Signal Transduct. Target. Ther. 10:76. doi: 10.1038/s41392-025-02145-7, PMID: 40059211 PMC11891338

[ref10] Kirkbride J. B. Anglin D. M. Colman I. Dykxhoorn J. Jones P. B. Patalay P. . (2024). The social determinants of mental health and disorder: evidence, prevention and recommendations. World Psychiatry 23, 58–90. doi: 10.1002/wps.21160, PMID: 38214615 PMC10786006

[ref11] Klotzbier T. J. Schott N. (2025). Scaffolding theory of maturation, cognition, motor performance, and motor skill acquisition (SMART COMPASS): a revised and comprehensive framework for understanding motor-cognitive interactions across the lifespan. Front. Hum. Neurosci. 19:1631958. doi: 10.3389/fnhum.2025.1631958 40874237 PMC12378464

[ref12] Laal M. (2011). Lifelong learning: what does it mean? Procedia Soc. Behav. Sci. 28, 470–474. doi: 10.1016/j.sbspro.2011.11.090

[ref13] Li J. Song D. Tong S. He Z. Qian J. Zhang Y. . (2025). Cross-sectional study of activity habits, socioeconomic status, and cognitive performance in Central China’s adult population. Sci. Rep. 15:15960. doi: 10.1038/s41598-025-00985-5, PMID: 40335565 PMC12059168

[ref14] Luo L. Jiang N. Zheng X. Wang P. Bi J. Xu F. . (2024). Effect of visual impairment on subjective cognitive decline in older adults: a cross-sectional study in China. BMJ Open 14:e072626. doi: 10.1136/bmjopen-2023-072626, PMID: 38688669 PMC11086556

[ref15] Ma H. Zhao H. Feng X. Gao F. (2025). The interaction between resilience framework and neuron-astrocyte-synapse dynamics in AD. Front. Aging Neurosci. 17:1644532. doi: 10.3389/fnagi.2025.1644532, PMID: 41019422 PMC12460312

[ref16] Maehler D. B. Martin S. Gorges J. Scherer R. (2025). Determinants of cognitive skills in adulthood: age cohort patterns. Int. J. Lifelong Educ. 44, 131–152. doi: 10.1080/02601370.2024.2390070

[ref17] Marzola P. Melzer T. Pavesi E. Gil-Mohapel J. Brocardo P. S. (2023). Exploring the role of neuroplasticity in development, aging, and neurodegeneration. Brain Sci. 13:1610. doi: 10.3390/brainsci13121610, PMID: 38137058 PMC10741468

[ref2000] Merton R. K. (1968). The Matthew effect in science: The reward and communication systems of science are considered. Science. 159, 56–63. doi: 10.1126/science.159.3810.56 5634379

[ref18] Morris T. P. Ai M. Chaddock-Heyman L. McAuley E. Hillman C. H. Kramer A. F. (2021). Relationships between enriching early-life experiences and cognitive function later in life are mediated by educational attainment. J. Cogn. Enhanc. 5, 449–458. doi: 10.1007/s41465-021-00208-5, PMID: 35005424 PMC8741175

[ref19] Nogueira J. Gerardo B. Santana I. Simoes M. R. Freitas S. (2022). The assessment of cognitive reserve: a systematic review of the most used quantitative measurement methods of cognitive reserve for aging. Front. Psychol. 13:847186. doi: 10.3389/fpsyg.2022.847186, PMID: 35465541 PMC9023121

[ref20] Oppl S. Stary C. (2020). Game-playing as an effective learning resource for elderly people: encouraging experiential adoption of touchscreen technologies. Universal Access Inf. Soc. 19, 295–310. doi: 10.1007/s10209-018-0638-0

[ref21] Reynolds C. F. 3rd Jeste D. V. Sachdev P. S. Blazer D. G. (2022). Mental health care for older adults: recent advances and new directions in clinical practice and research. World Psychiatry 21, 336–363. doi: 10.1002/wps.20996, PMID: 36073714 PMC9453913

[ref22] Thwe W. P. Kalman A. (2024). Lifelong learning in the educational setting: a systematic literature review. Asia Pac. Educ. Res. 33, 407–417. doi: 10.1007/s40299-023-00738-w, PMID: 41159878

[ref23] Tohit N. F. M. Haque M. (2024). Preparing the younger generation for an aging society: strategies, challenges, and opportunities. Cureus 16:e64121. doi: 10.7759/cureus.64121 38983672 PMC11231670

[ref24] Uno A. Browne R. Shinada T. Takahashi M. Soga K. Du Y. . (2025). Effects of a digital visual art learning intervention in healthy older adults: a pilot randomized controlled trial. Front. Aging 6:1635789. doi: 10.3389/fragi.2025.1635789, PMID: 40977990 PMC12444092

[ref25] Yamashita T. López E. B. Stevens J. Keene J. R. (2017). Types of learning activities and life satisfaction among older adults in urban community-based lifelong learning programs. Activities Adapt. Aging 41, 239–257. doi: 10.1080/01924788.2017.1310583

[ref26] Zou H. Zhang S. Cui X. Xu H. Zhou Z. Cheng D. . (2025). Advancements in the investigation of the mechanisms underlying cognitive aging. Biogerontology 26:158. doi: 10.1007/s10522-025-10300-4, PMID: 40783909 PMC12336085

