# Supplementary material for: Lifelong learning dimensions and their associations with late-life cognitive decline: moderating roles of socioeconomic status and early life education
Source: Front Psychol. 2025 Nov 11;16:1729306. doi: 10.3389/fpsyg.2025.1729306 (PMC12644077; doi:10.3389/fpsyg.2025.1729306)
Supplement: Supplementary file 1 [file Data_Sheet_1.pdf]

Questionnaire on Lifelong Learning and Cognitive Health in Old Age

Dear elders and their assistants:
Hello!
We are a research team from the School of Ethnology and Sociology, Minzu University of China, and we are conducting a study on the relationship between learning activities and cognitive health among older adults. We sincerely invite you to participate in this questionnaire.
This questionnaire aims to understand the older adults' learning and living habits, as well as their self-perceptions of cognitive status in old age. Your valuable answers will help us better understand how to promote physical and mental health in old age through learning, and provide a basis for formulating more effective elderly care policies and health intervention measures.
Please note:
The main respondent of this questionnaire is you (the older adult). Please answer the questions based on your true feelings and actual situations.
If you (the older adult) have difficulties in reading or writing, please ask your family member (assistant) to read the questions to you and record your (the older adult) answers.
To assistants (family members):
Thank you for assisting your family member (the older adult) to complete this questionnaire. To ensure the authenticity and accuracy of the data, we earnestly request you to:
Read each question and all options in the questionnaire word by word without skipping any part.
Please ensure that the older adult understands each question and provides their own answer.
Please do not explain, guide, or give any hints about the questions, nor answer on behalf of the older adult.
Please faithfully record the older adult's own answers regardless of whether you agree with them or not.
Please allow the older adult sufficient time to think without rushing.
During the filling process, please try to maintain a quiet and undisturbed environment.
Your cooperation is crucial for this research. Thank you very much for your support and understanding!
This questionnaire is expected to take 20-30 minutes to complete. All information will be strictly confidential and only used for academic research. Please feel free to fill it out.
Module 1: Informed Consent and Basic Information (Confirmed by the older adult themselves or the assistant)
Important Notice: Please ask the assistant to read the following consent statement aloud to the older adult.
Informed Consent: I have read and understood the purpose, procedures, and confidentiality commitment of this research, and I know that my participation is completely voluntary. I agree to participate in this research and provide true information.
Yes (The older adul agrees)
No (The older adult does not agree)
(If "No" is selected, the questionnaire will terminate)
Please answer the following questions according to the older adult's actual situation.
1. What is your age?
60 - 64 years old
65 - 69 years old
70 - 74 years old
75 - 79 years old
80 years old and above
2. What is your gender?
Male
Female
3. What is your marital status?
Unmarried
Married/Cohabiting

Divorced
Widowed
4. What is your ethnicity?
Han
Ethnic Minority (Please specify: _______)
5. Where do you currently live?
City (Municipality directly under the Central Government, provincial capital city, or prefecture-level city)
Town (County seat or township)
Village (Rural area)
6.What is your current living situation?
Living alone
Living with spouse/partner
Living with children
Living with other relatives
Nursing home
Other (Please specify:_____)
Module 2: Early Life Education (ELE) Details
Assistant's prompt: Please read the following questions carefully to ensure that the older adult understand and answer based on their own recollection.
7.How many years of formal schooling have you received in total? (For example: 6 years of primary school, 3 years of junior high school, 3 years of senior high school, 4 years of university, then it is 16 years)
0 years (No education)
1 - 6 years (Primary school and below)
7 - 9 years (Junior high school)
10 - 12 years (High school/Secondary vocational school/Technical school)
13 - 15 years (Junior college)
16 years or above (Bachelor's degree or above)
8. What is the highest level of education you have completed?
No education
Primary school Junior high school
High school/Secondary vocational school/Technical school
Junior college
Bachelor's degree
Master's degree or above
9. When you were a child (in primary and junior high school), were there many books in your family? (For example: apart from textbooks, how many extracurricular books, magazines, etc.)
Very few, almost none
Relatively few
Average
Relatively many
Very many
10. When you were a child, did your parents or family encourage you to study, read, or participate in some extracurricular activities (such as attending tutoring classes, learning musical instruments, etc.)?
Never
Rarely
Sometimes
Often
Always
11. When you were a child, did you have the opportunity to learn a foreign language or a musical instrument?
Yes
No
Module 3: Socioeconomic Status (SES) Assessment
Assistant's prompt: Please read the following questions carefully to ensure that the elderly understand and answer from their own memory.

12. What was your occupation before retirement or what is your current occupation? (Please ask the elderly to describe and the assistant record the closest option)
No occupation/Farming/Housework
Ordinary worker/Waiter/Peddler
General clerk/Technician/Primary school teacher
Middle-level manager/Engineer/Doctor/Middle school teacher
Senior manager/Professor/Expert/Entrepreneur
Other (Please specify:_____)
13. What is the approximate average monthly total income of your current family? (Refers to the total income of all family members, including pensions, subsidies from children, etc.)
Less than 3000 yuan
3001 - 5000 yuan
5001 - 8000 yuan
8001 - 12000 yuan
More than 12001 yuan
14. What is the property right of the house you currently live in?
Self-owned property (completely belonging to oneself or family members)
Rented housing (renting a house from a unit or an individual)
Unit - allocated housing (without property rights or with partial property rights)
Others (please specify:_____)
15. Which level do you think your family's economic situation is in the local society?
Lower
Lower - middle
Medium
Upper - middle
Higher
Module 4: Lifelong Learning Participation (LLL) Assessment
Assistant's Prompt: Please read the following questions carefully. Ask the elder to recall whether he/she has participated in the following activities in the past year and the frequency of participation. Please let theolder adult answer by himself/herself.
16.Please choose the option that best matches your frequency of participation according to your actual situation in the past year:

Frequency of learning activities per week	1 = Never	2 = Occasionally	3 = A few times a month	4 = Every	5 = Every day
Read books, newspapers, magazines or online articles (not for work)?	[ ]	[ ]	[]	[]	[]
Attend various courses, lectures or training (calligraphy, painting, singing, dancing, computer, foreign language, health lectures, etc.)	[]	[]	[]	[]	[]
Learning new skills or hobbies? (e.g. cooking, gardening, crafts, photography, musical instruments etc.)	[]	[]	[]	[ ]	[]
Participate in volunteer activities or community services?	[]	[]	[]	[]	[]
Use smartphones, tablets or computers to learn new knowledge, or communicate with people through the Internet?			[]	[ ]	[]
Have in-depth and thoughtful discussions or debates with others?	[]	[ ]	[]	[]	[]
Travel and actively learn about new cultures, histories or geographical knowledge?	[]	[]	[]	[]	[]
Play puzzle games? (such as mahjong, chess, sudoku, jigsaw puzzles, bridge, etc.,	[]	[]	[]	[ ]	[]

excluding gambling nature)					
Going to museums, art galleries, libraries, science and technology museums and other cultural places?	[]	[]	[]	[]	[]
Watching documentaries, educational programs or science videos to learn new things?	[]	[]	[]	[]	[]

Module 5: Subjective Cognitive Function (SCF) Assessment

Assistant's Prompt: Please read the following questions carefully. Ask the older adult to choose the option that best reflects his/her true feelings in the past month. Please let the older adult answer by himself/herself.

17. Please choose the option that best suits your situation based on your feelings in the past month:

Description of cognitive feelings	1 = Never	2 = Seldom	3 = Sometimes	4 = Often	5 = Always
Do you feel that your memory is not as good as it was 5 years ago?	[]	[]	[]	[]	[]
Do you find it more difficult to learn new things than before?	[]	[]	[]	[]	[]
Do you have difficulty concentrating in your daily life (such as making a shopping list, arranging a schedule)?	[]	[ ]	[]	[ ]	[]
Do you find yourself more likely to forget what you've just said or done than before?	[]	[]	[]	[]	[]
Do you feel more strained than before when solving complex problems or making decisions?	[]	[]	[]	[]	[]
Do you have difficulty finding the right words to express yourself or does it take you longer?	[]	[]	[]	[]	[]
Do you feel that you are less efficient at doing things than before?	[]	[]	[]	[]	[]
Have you ever walked into a room and forgotten what you were going to do?	[]	[]	[]	[]	[]

Module 6: Health Status and Lifestyle (Control Variables)

Assistant's Prompt: Please read the following questions carefully to ensure the elder understands and answers based on their own recollection.

18. Do you currently have any of the following chronic diseases? (Multiple choices are possible)

Hypertension
Diabetes
Heart disease (such as coronary heart disease, angina pectoris, etc.)
Stroke/Cerebrovascular accident (including transient ischemic attack TIA)
Depression or other mental illnesses (diagnosed and under treatment)
Parkinson's disease
Alzheimer's disease/Dementia (diagnosed)
Other (Please specify:_____)
No diseases mentioned above

19. How many times do you engage in moderate-intensity physical activities per week? (For example: brisk walking, square dancing, tai chi, etc., at least 30 minutes each time)

0 times
1 - 2 times
3 - 4 times
5 times and above

20. Do you smoke?

Never smoked
Have quit smoking
Smoke occasionally (a few times a month or less)
Smoke frequently (a few times a week or daily)

21. Do you drink alcohol?
Never drink alcohol
Have quit drinking
Drink occasionally (a few times a month or less)
Drink frequently (a few times a week or daily)
22. How many hours do you sleep on average every night?
Less than 5 hours
5 - 6 hours
7 - 8 hours
9 hours or more
23. What is your current eyesight condition? (corrected eyesight after wearing glasses)
Very good
Good
General
Poor
Very poor
24. What is your current hearing condition? (Corrected hearing after wearing hearing aids)
Very good
Good
General
Goor
Very poor
Module 7: Assistant Information (Filled in by the assistant)
Assistant's Reminder: Please answer the following questions yourself (the assistant).
25. What is your relationship with the elderly person being surveyed?
Spouse
Child
Grandchild
Other relative
No blood relation, friend/community volunteer/caregiver, etc.
26. What is your highest level of education?
Primary school or below
Junior high school
High school/technical secondary school/vocational school
Junior college
Bachelor's degree
Master's degree or above
27. What is your approximate age?
18 - 30 years old
31 - 45 years old
46 - 60 years old
61 years old and above

This is the end of the questionnaire. Thank you again (the older adults) and you (the assistant) for your precious time and support!
